# ODAD1 variants resulting from splice-site mutations retain partial function and cause primary ciliary dyskinesia with outer dynein arm defects

**DOI:** 10.3389/fgene.2023.1270278

**Published:** 2023-10-31

**Authors:** Nannan Zhou, Weilin Liang, Yanzhu Zhang, Guoli Quan, Ting Li, Siqing Huang, Yating Huo, Haiyan Cui, Yuanxiong Cheng

**Affiliations:** Department of Pulmonary and Critical Care Medicine, The Third Affiliated Hospital of Southern Medical University, Guangzhou, China

**Keywords:** CCDC114, *ODAD1*, outer dynein arm, outer dynein arm docking complex, primary ciliary dyskinesia, truncated protein

## Abstract

Primary ciliary dyskinesia (PCD) is a genetically heterogeneous disorder caused by defects in motile ciliary function and/or structure. *Outer dynein arm docking complex subunit 1* (*ODAD1*) is an important component of the outer dynein arm docking complex (ODA-DC). To date, 13 likely pathogenic mutations of *ODAD1* have been reported. However, the pathogenesis of *ODAD1* mutations remains elusive. To investigate the pathogenesis of splice-site mutations in *ODAD1* discovered in this study and those reported previously, molecular and functional analyses were performed. Whole-exome sequencing revealed a compound mutation in *ODAD1* (c.71-2A>C; c.598-2A>C) in a patient with PCD, with c.598-2A>C being a novel mutation that resulted in two mutant transcripts. The compound mutation in *ODAD1* (c.71-2A>C; c.598-2A>C) led to aberrant splicing that resulted in the absence of the wild-type ODAD1 and defects of the outer dynein arm in ciliary axonemes, causing a decrease in ciliary beat frequency. Furthermore, we demonstrated that the truncated proteins resulting from splice-site mutations in *ODAD1* could retain partial function and inhibit the interaction between wild-type ODAD1 and ODAD3. The results of this study expand the mutational and clinical spectrum of PCD, provide more evidence for genetic counseling, and offer new insights into gene-based therapeutic strategies for PCD.

## 1 Introduction

Primary ciliary dyskinesia [PCD (MIM 244400)] is a genetic disease caused by defects in motile ciliary function and/or structure. The global prevalence of PCD is estimated to be at least 1 in 7,554 individuals (Hannah et al., 2022). PCD is usually inherited in an autosomal recessive manner (Legendre et al., 2021; Mitchison and Smedley, 2022); however, cases with autosomal dominant (Wallmeier et al., 2019; Legendre et al., 2021) and X-linked (Moore et al., 2006; Legendre et al., 2021) inheritance have also been reported. Clinical features of PCD include recurrent pulmonary infection, chronic sinusitis and otitis media, situs inversus, and infertility (Zariwala et al., 2019). PCD not only severely reduces the quality of life and life span of patients but also significantly increases the levels of anxiety and depression in patients and their caregivers (Verkleij et al., 2021).

The axoneme structure of the human motile cilium consists of a pair of central microtubules surrounded by nine peripheral outer doublet microtubules (DMTs, A tubule and B tubule). Four identical outer dynein arms (ODA) and six different inner dynein arms (IDA) are arranged in two rows on the A tubule of DMTs. They slide along the B tube, causing the cilium to beat (Ishikawa, 2017; Legendre et al., 2021). *Outer dynein arm docking complex subunit 1* (*ODAD1*) encodes an approximately 79-kDa protein containing six coiled-coil domains. ODAD1 is an important part of the outer dynein arm docking complex (ODA-DC), which is essential for the correct assembly of the ODA in ciliary axonemes (Gui et al., 2021; Braschi et al., 2022). In 2013, (Onoufriadis et al., 2013; Knowles et al., 2013), were the first to report that *ODAD1* mutations can cause PCD in humans. In the following decade, *ODAD1* was extensively investigated (Li et al., 2019; Chen et al., 2020; Guo et al., 2020; Alsamri et al., 2021; Guan et al., 2021; Hannah et al., 2022; Ostrowski et al., 2022). However, to date, most studies on *ODAD1* have implemented statistical analysis of clinical data from patients with PCD and suggested that mutations in *ODAD1* may lead to the corresponding mRNA changes and eventually cause PCD. Moreover, studies on the pathogenesis of *ODAD1* mutations are lacking.

At present, no therapeutic strategies are available to restore ciliary function in patients with PCD, and gene therapy is the most promising treatment. However, gene therapies based on lentiviral vectors, lipid nanoparticles, and gene editing are difficult to apply to clinical practice because of their poor therapeutic effects and severe side effects (Chhin et al., 2009; Ostrowski et al., 2014; Lai et al., 2016; Woo et al., 2022). Therefore, identifying specific PCD genotypes and understanding the mechanisms underlying disease development are primary approaches to developing individualized treatment and restoring ciliary function.

In this study, whole-exome sequencing revealed a compound heterozygous mutation in *ODAD1* (c.71-2A>C; c.598-2A>C) in a patient with typical clinical symptoms of PCD. Of these two mutations, c.598-2A>C is a novel pathogenic mutation. Molecular, biochemical, and functional analyses were performed to investigate the pathogenesis of splice-site mutations in *ODAD1* discovered in this study and those reported previously.

## 2 Material and methods

### 2.1 Patient information and sample collection

A 12-year-old girl with situs inversus, recurrent respiratory infections and chronic rhinosinusitis, her parents, and non-PCD controls (6 individuals) were included in this study. Based on clinical symptoms, a decrease in ciliary beat frequency by high-speed video microscopy, ciliary structural defects detected by transmission electron microscopy, and exome sequencing, the patient was diagnosed with PCD. The six control subjects were excluded from the diagnosis of PCD and acute or chronic airway inflammation. The collection and use of peripheral blood, respiratory epithelial cells and bronchial biopsy specimens were approved by the Ethical Review Board of the Third Affiliated Hospital of Southern Medical University, and written consent was obtained. Clinical data were collected using standardized methodologies as reported previously (Shapiro et al., 2016; Zariwala et al., 2019; Guan et al., 2021).

The blood samples of the proband, her patrents and non-PCD controls (3 individuals) were used for DNA and RNA extraction.

Respiratory epithelial cells were obtained by segmental bronchus brushing of the proband and the controls (3 individuals) with a cytological soft sterile brush (AF -D1810WA, Alton, China). Each subject brushed once with a back and forth motion for a total of 8–10 strokes. Cells were suspended in PneumaCult-Ex Plus Medium (Stem cell, 05040) prewarmed to 37°C and used for high-speed video microscopy (HSVM) analysis. Then, bronchial biopsy specimens were collected from the segmental bronchus of the proband and the controls (3 individuals) using a disposable biopsy sampling forceps (AF -D1810BT, Alton, China). Three biopsies were taken from each subject. One was used to extract RNA for RNA analysis, one was fixed in 4% paraformaldehyde for immunofluorescence, and one was fixed in 2.5% glutaraldehyde for transmission electron microscopy.

### 2.2 Whole-exome sequencing

Whole-exome sequencing was performed as described previously (Knowles et al., 2013; Onoufriadis et al., 2013). Genomic DNA was extracted from the peripheral blood and fragmented using Covaris S220 (Covaris, USA), with an average size of 150bp. The exonic DNA sequences were enriched using the GenCap V4.0 probe sequence capture array (Mygenostic, China), which captures the exons and the intron regions adjacent to the exons (50 bp in size). Sequencing was performed using the DNBSEQ-T7 (MGI Tech CO., Ltd, China). The final targeted region was 49.11 Mb and included 23,000 genes. The average sequencing depth was 185.59; 98.65% of target bases were covered at 10×, and 97.56% of target bases were covered at 20×. The raw data were assessed for quality using the base-calling method according to manufacturer’s instructions. The remaining sequence reads were aligned to the human reference genome (GRCh38/hg38 and GRCh37/hg19) using Burrows Wheeler Aligner (BWA; Version 0.7.16). Then the variants were further annotated using Variant Effect Predictor (http://asia.ensembl.org/info/docs/tools/vep/index.html) and ANNOVAR (http://www.openbioinformatics. org/annovar) and compared with 1,000 Genome Project, ESP6500SI, dbSNP, EXAC_ALL, HGMD and ClinVar. The pathogenicity of the candidate variants was evaluated based on the American College of Medical Genetics and Genomics guideline (ACMG). In addition, PCR amplification and Sanger sequencing were further performed to validate the candidate variants and segregation analyses were performed in family members. The primers used for PCR and Sanger sequencing are listed in Supplementary Tables S3, S4.

### 2.3 Nasal nitric oxide (nNO) measurement

Nasal NO was measured with an EcoMedics CLD88 NO analyzer (Duernten, Switzerland). Because the individuals in this study were older than 5 years and were able to cooperate well, the nNO measurement was performed by exhalation against resistance maneuver as described previously (Leigh et al., 2013; Shapiro et al., 2020). The individuals had been in her baseline state of health for at least 2 weeks before the nNO measurement. The technician monitored the nNO curve on a computer screen and required an exhalation time of up to 30 s to achieve an acceptable plateau phase of at least 3 s that had less than 5% deviation. This might require several attempts until two successful trials are achieved in the first nostril. Then the procedure was repeated in the contralateral nostril, and two more plateau measurements were made in the same way. The plateau value in the same nostril should be less than 10%, and the plateau value between the right and left nostrils should be similar under ideal conditions. However, due to the difference in nasal airflow, the nNO values of the left and right nostril might differ by more than 10%. If the difference between the left and right nostrils was more than 10%, repeated the measurement at least twice in the same phase in each nostril. Even if there was such a difference, the maximum repeatable value of each nostril should be reported. The nNO production (nL/min) was calculated using the following equation: nNO (nL/min) = NO (ppb) × sampling rate (mL/min).

### 2.4 RNA splicing analysis

To evaluate the expected splicing results of the *ODAD1* mutations, the SpliceAI model (v1.3.1) was applied to two sequences (reference allele and variant allele), calculating the probability of each nucleotide being used as an acceptor or donor site in a biological context. Prediction results are then visualized using SpliceAI-visual (https://mobidetails.iurc.montp.inserm.fr/MD/) as previously described (Agathe et al., 2023). The reference genome version was GRCh38/hg38.

To determine the effects of *ODAD1* mutations on transcripts, reverse transcription polymerase chain reaction (RT-PCR) was performed using RNA extracted from the bronchial mucosa biopsy specimens of patient Ⅲ:1 and control individuals according to the manufacturer’s protocol. The primers used for PCR had the corresponding restriction enzyme sites (Supplementary Table S3). The amplification fragment was cloned into the pB-CAG-Amp vector to construct a recombinant plasmid containing *ODAD1* cDNA fragment and was identified via Sanger sequencing (Figures 2A, C). The primers used for Sanger sequencing are listed in Supplementary Table S4.

### 2.5 Prediction of the protein structure

To investigate the effects of *ODAD1* mutations on the tertiary structure of ODAD1 protein, we predicted the tertiary structures of six ODAD1 variants: two (mutants 1, 2) identified in this study and four (mutants 4–7) reported in previous studies (Knowles et al., 2013; Onoufriadis et al., 2013; Ostrowski et al., 2022) (Supplementary Table S2). Briefly, the primary structures of proteins were predicted using SnapGene (version 3.2.1) based on the base sequences of *ODAD1* mutant transcripts. Subsequently, three-dimensional models were constructed using SWISS-MODEL (https://swissmodel.expasy. org/interactive#structure, A0A6I8PTZ2) and optimized using the Pymol software.

### 2.6 Immunofluorescence analysis

Bronchial mucosa biopsy samples collected from patient Ⅲ:1 and control individuals were fixed, dehydrated, embedded, and cut into 10-μm-thick cryosections. Thereafter, the sections were incubated with mouse monoclonal anti-acetylated tubulin antibody (Sigma, T7451) and rabbit polyclonal anti-ODAD1 antibody (Novus, NBP1-93863) overnight at 4°C. The following day, the sections were incubated with Anti-mouse Alexa Fluor 488 antibody (Abcam, ab150105) and Dnk pAb to Rb IgG Alexa Fluor 594 antibody (Abcam, ab150064) and stained with DAPI (Yeasen, 36308ES11). Images were acquired using an Olympus FV3000 confocal microscope (Olympus, Japan).

### 2.7 Transmission electron microscopy

Bronchial biopsy specimens were immersed in 2.5% glutaraldehyde immediately after collection. The following day, the specimens were fixed in 1% osmium tetroxide, dehydrated in a graded ethanol series, and embedded in epoxy resin. Subsequently, the specimens were cut into 60-nm-thick sections and stained with 2% methanolic uranyl acetate. Finally, the ultrastructure of cilia was analyzed using a transmission electron microscope (JEM -1,400; Jeol, Tokyo, Japan).

### 2.8 Measurement of ciliary beat frequency

Respiratory epithelial cells were collected via bronchial brush biopsy and suspended in PneumaCult-Ex Plus Medium (Stem cell, 05040) preheated to 37°C. Ciliary activity was recorded at 120 frames/second using an inverted microscope (MI52-M; Mingmei, Guangzhou, China) as described previously (Knowles et al., 2013; Onoufriadis et al., 2013). To evaluate ciliary beat frequency (CBF), digital recordings were evaluated by three investigators in a blinded fashion. We used slow-motion playback of the video sequence to generate tracings of the ciliary beat. At least 3 visual fields per sample and 10 ciliary edges per visual field were traced manually.

### 2.9 Construction of expression vectors

Plasmids containing the wild-type sequence of *ODAD1* and the mutant C-terminal fragments of *ODAD1* (Mutant1, Mutant4 and Mutant6) was obtained from Azensta (Guangzhou, China). The cDNA of every mutant *ODAD1* (mutants 1-7) with *V5* tag was obtained with position-specific primers carrying corresponding restriction enzyme sites by PCR (Vazyme, P520- AA) and T4 ligase (Takara, 2011A).

To obtain the cDNA of *outer dynein arm docking complex subunit 3* (*ODAD3*), RNA was extracted from bronchial mucosa biopsies using Trizol (Invitrogen, 10296010) and first strand complementary DNA was synthesized with Sensiscript RT kit (QIAGEN, 205213). PCR was performed with position-specific primers carrying corresponding restriction enzyme sites and 2×Phanta flash Master Mix (Vazyme, P520- AA) according to the manufacturer’s protocols.

To generate the expression vectors of pb-CAG-ODAD1 WT-V5-IRES-BSD-T2A-BFP, pb-CAG-ODAD1 Mutant-V5-IRES-BSD-T2A-BFP, V5-tagged wild-type *ODAD1* and *ODAD1* mutants 1-7 were cloned into a piggyBac transposable backbone carrying an IRES- BSD-T2A-BFP element. And meanwhile, *ODAD3* was cloned into a piggyBac transposable backbone carrying a 3×HA- IRES- Puro-T2A-GFP to generate the pb-CAG-ODAD3-HA-IRES-Puro-T2A-GFP vector (Figure 5A).

All wild-type cDNA clones were confirmed by Sanger sequencing and matched RefSeq gene accession number NM_001364171 (*ODAD1*) or NM_145045 (*ODAD3*). The *ODAD1* mutant clones were confirmed by sequence analysis and matched with the results of mutant transcripts we discovered (mutants 1, 2) and previously published (mutants 4-7). The primers used for cDNA cloning and Sanger sequencing are listed in Supplementary Tables S3, S4.

### 2.10 HEK293FT cell culture and transfection

Given the advantages of 293FT cells, such as rapid proliferation, high transfection efficiency, and the structure of the expressed protein closest to its conformation in the human body, we chose 293FT cells to study the interaction between ODAD1 and ODAD3. The HEK293FT cell line was a generous gift from Xuefei Gao (Basic Medical College of Southern Medical University). The cells were cultured in DMEM supplemented with 10% FBS (Gibco, 10099141C) and 1% penicillin and streptomycin (Gibco, 15140122) at 37°C.

To examine the interaction between ODAD1 variants and ODAD3, HEK293FT cells were co-transfected with plasmids encoding V5-tagged wild-type or mutant *ODAD1* or empty plasmids and plasmids encoding HA-tagged *ODAD3* through electro-transfection (Lonza, Switzerland) according to the manufacturer’s protocol (Figure 5B).

To assess whether mutant ODAD1 affects the interaction between wild-type ODAD1 and ODAD3, vector encoding C-terminal V5-tagged mutant *ODAD1* or empty plasmid was co-transfected with vector encoding C-terminal V5-tagged wild-type *ODAD1* and HA-tagged *ODAD3* in 293FT cells. The empty plasmid was used as a control (Figure 6A). The total amount of each plasmid used was 2 μg. The cells were screened in medium containing BSD (10 μg/mL) and Puro (1 μg/mL). The construction of expression vectors is described in Supplemental files.

### 2.11 Co-immunoprecipitation and immunoblotting

Proteins extracted from HEK293FT cells were incubated with 4 µg of HA-tagged polyclonal antibody (Invitrogen, 71-5,500) and 4 µg of rabbit IgG (Cell Signaling Technology, 2729s) overnight at 4°C. The following day, each sample was incubated with 20 μL of DynabeadsTM Protein G (Invitrogen, 91216624) on a shaker for 2 at 4°C to capture immunoprecipitates. Bead complexes were washed thrice with IP lysis buffer (Beyotime, P0013), resuspended in a sample buffer (0.5% PMSF, 1% PI, 2% TCEP, and 25% SDS loading in IP lysis buffer), and heated for 10 min at 70°C.

Immunoblotting was performed as described previously (Zheng et al., 2022). The loading amount of Input was 20μg, and the volume of IgG and IP used was 12 μL. Membranes were incubated with V5-tag rabbit PolyAb (Proteintech, 14440-1-AP) and HA-tag rabbit mAb (Cell Signaling, C29F4) at 4°C overnight and with IPKine™ HRP Mouse Anti-Rabbit IgG LCS (Abbkine, A25022) at RT for 1 h. Images were digitally acquired using Amersham Imager 600 (Cytiva,USA). The expression of different proteins was quantified using the ImageJ software.

### 2.12 Statistical analysis

All statistical analyses were performed using the GraphPad Prism software (version 9.00). Data were expressed as the mean ± standard deviation. A *t*-test with two-tailed distribution was used to compare the data of two groups, whereas one-way analysis of variance (ANOVA) was used to compare the data of three or more groups. A *p*-value of<0.05 was considered significant.

## 3 Results

### 3.1 Patients with typical characteristics of PCD had a compound mutation in *ODAD1*

**FIGURE 1:**
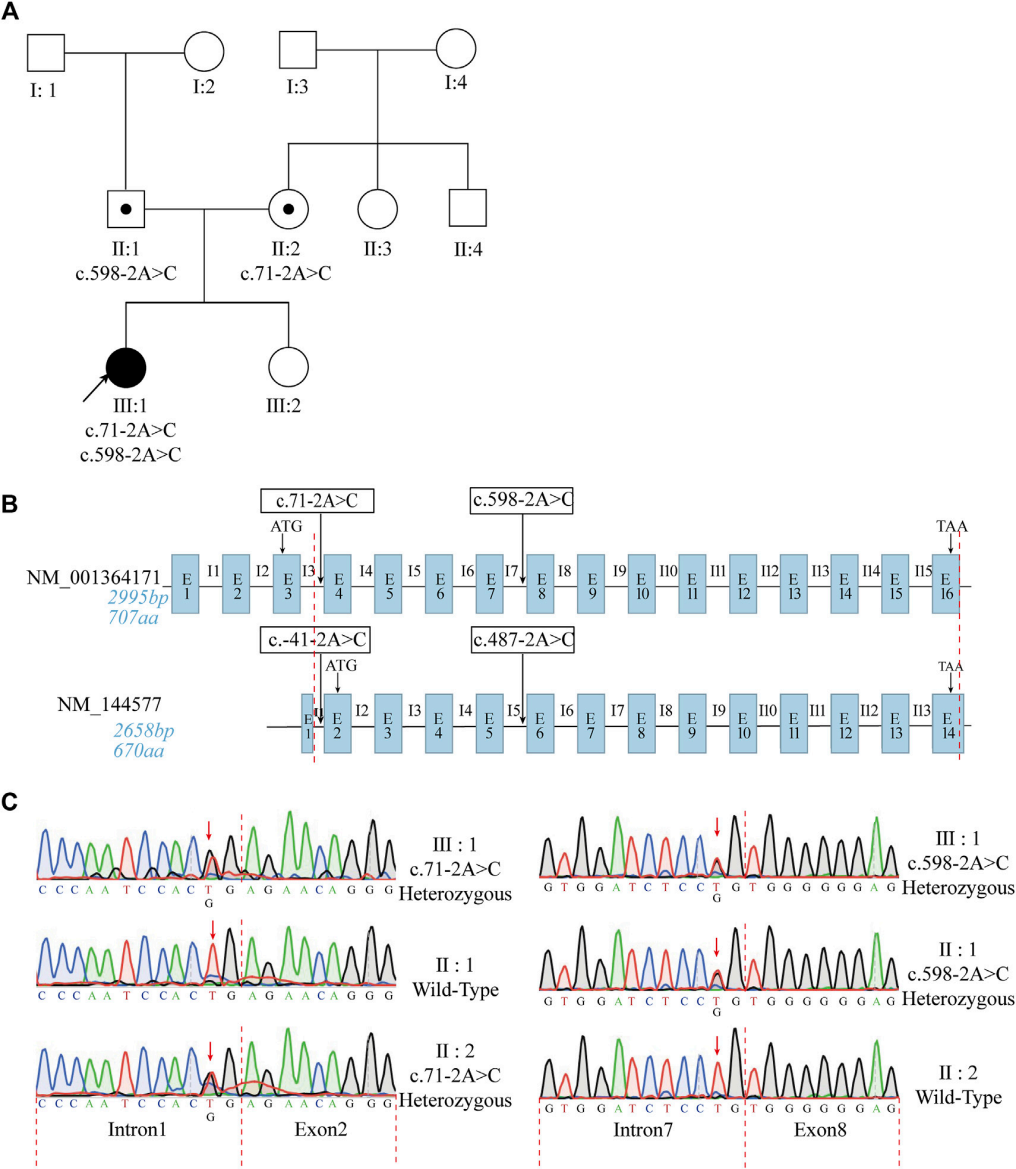
The patient with typical characteristics of PCD had a compound mutation in *ODAD1*. **(A)** Pedigree of the patient’s family. The black arrow indicates the proband. **(B)** Structural similarities and differences between the two transcripts of *ODAD1*. The transcript NM_001364171 consists of 2,995 bp and contains 16 exons encoding a protein with 707 amino acids. The transcript NM_144577 consists of 2,658 bp and contains 14 exons encoding a protein with 670 amino acids. The base sequences between the red dotted lines are identical. The start and stop codons are indicated. The positions of the identified *ODAD1* mutations are indicated. In-trons/exons are not drawn to scale. E, exon; I, intron. **(C)** Sanger sequencing revealed a compound mutation in the patient and parents, who harbor the respective mutations. The red arrow indicates the mutation sites.

The proband (patient Ⅲ:1) included in this study is the only patient in the Chinese family (Figure 1A; Supplementary Table S1). She developed a productive cough, chronic sinusitis, and otitis media as a young child. Besides, her nNO value was low (5.4 nL/min), and pulmonary function test revealed severe obstructive ventilator dysfunction (Supplementary Table S1). Computed tomography (CT) and magnetic resonance imaging (MRI) confirmed bronchiectasis, situs inversus totalis, and pansinusitis (Supplementary Figure S1).

Previously, all researchers except Ostrowski et al. (2022) have used NM_144577 in studies on *ODAD1* (Knowles et al., 2013; Onoufriadis et al., 2013; Li et al., 2019; Chen et al., 2020; Guo et al., 2020; Guan et al., 2021; Hannah et al., 2022; Kos et al., 2022). In this study, NM_001364171 was detected in bronchial mucosa biopsy samples and peripheral blood cells collected from patient Ⅲ: 1 and control individuals (Supplementary Figure S2). This finding is consistent with that of a study by Ostrowski et al. (2022). (Ostrowski et al., 2022). Therefore, the transcript NM_001364171 was selected for further analysis. Structural similarities and differences between the two *ODAD1* transcripts and the mutation sites of *ODAD1* are shown in Figure 1B.

High-throughput exon-sequencing was used to identify candidate genes related to the pathogenesis of PCD. The results revealed two mutations in *ODAD1*, including c.71-2A>C and c.598-2A>C (Figure 1; Table 1). The results of Sanger sequencing demonstrated that c.71-2A>C and c.598-2A>C mutations were inherited from the mother and father, respectively (Figure 1C; Table 1; primer sequences are shown in Supplementary Tables S3, S4).

**TABLE 1:** *ODAD1* mutations in the patient with PCD and her parents.

ID	Gene	Chromosomal location (hg38)	Transcript	Exon/Intron	Base change	SpliceAIDS (DP)^a^	cDNA transcript after RT-PCR^b^	Predicted amino acid change	Zygosity	Segregation^c^	ACMG guideline
Ⅲ:1	*ODAD1*	chr19: g.48318814	NM_001364171	Intron3	c.71-2A>C	AG = 0.03 (−21)	uncertain	uncertain	Heterozygous	Maternal	Pathogenic PVS1+PM2+PM3
						AL = 0.68 (−2)					
						DG = 0.00 (−16)					
						DL = 0.00 (−2)					
Ⅲ:1	*ODAD1*	chr19: g.48306325	NM_001364171	Intron7	c.598-2A>C	AG = 0.00 (−31)	r.598_665del,r.598_1598-59ins; 598-665del	p.Glu200Glyfs*60,p.Glu200_Val221delins	Heterozygous	Paternal	Pathogenic PVS1+PM2+PM3
						AL = 0.96 (−2)					
						DG = 0.00 (38)					
						DL = 0.13 (−25)					

^a^ SpliceAI: delta score (DS), delta position (DP), acceptor gain (AG), acceptor loss (AL), donor gain (DG), donor loss (DL).

^b^ Primer sequences in this study were listed in Supplementary Table S3. RT-PCR, was carried out on biopsy tissues of human bronchial mucosa.

^c^ Mutant allele shown to segregate with either the father’s (paternal) or mother’s (maternal) side of the family.

### 3.2 Mutations in *ODAD1* (c.71-2A>C; c.598-2A>C) resulted in aberrant splicing

To analyze the effects of *ODAD1* mutations on the resulting transcripts, silico predictions were performed. The results showed that c.71-2A>C weakened the canonical acceptor site in Intron 3 (acceptor loss of 0.68), and c.598-2A>C weakened the canonical acceptor site in Intron 7 (acceptor loss of 0.96) (Table 1; Supplementary Figure S3).

**FIGURE 2:**
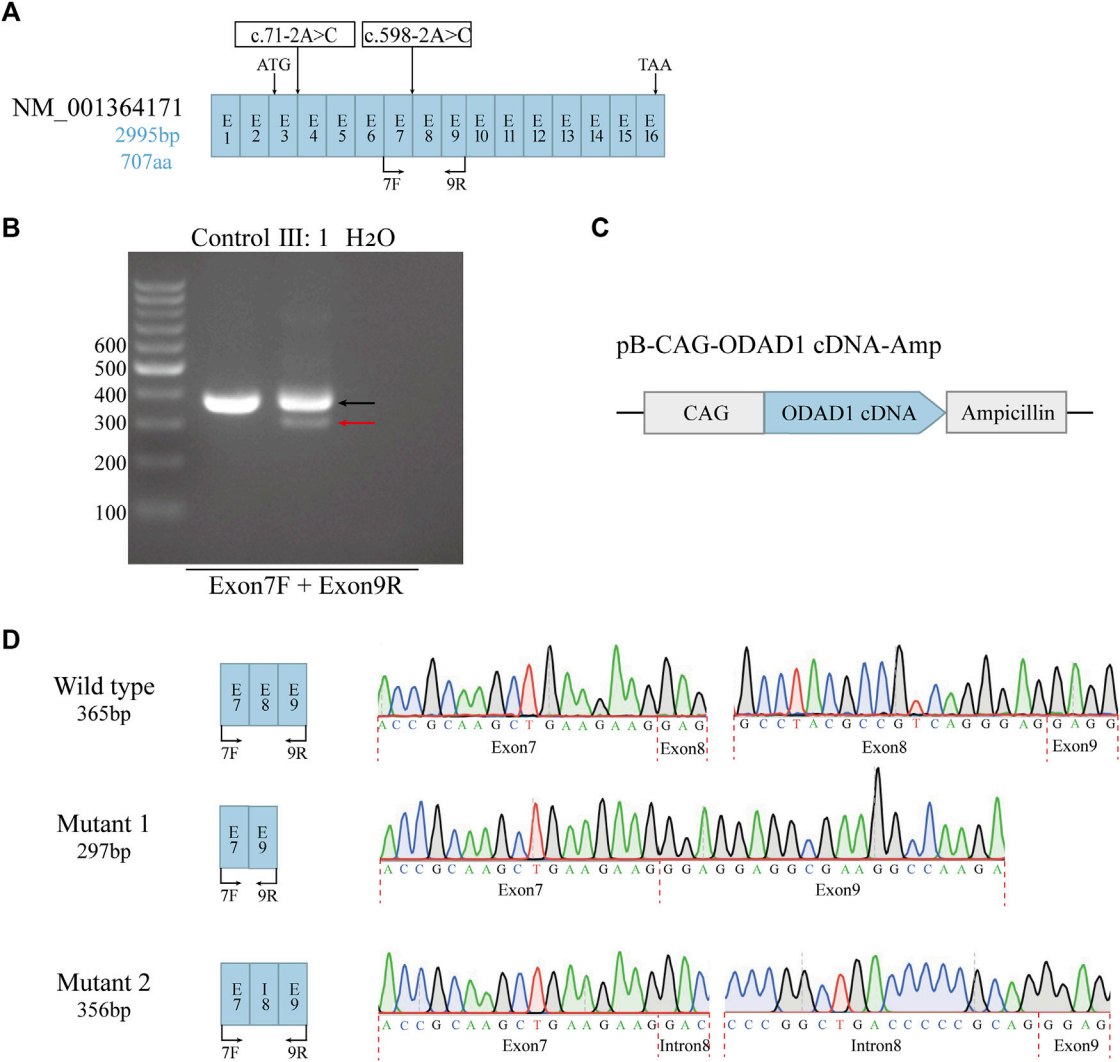
The effects of mutations on *ODAD1* transcripts were examined via reverse transcription PCR. **(A)** Structural diagram of the NM_001364171 transcript. The start and stop codons are indicated. The positions of the identified *ODAD1* mutations are indicated. Exons are not drawn to scale. **(B)** The primer set (Exon7F + 9R) showed that patient Ⅲ:1 had two different amplification products. One was similar in size to the control (indicated by the black arrow), and one was smaller than the control (indicated by the red arrow). **(C)** Genomic maps of plasmids used in the study. CAG is composed of the enhancer sequence of the CMV promoter, the chicken-β-actin promoter, and the rabbit-β-globin splice receptor. **(D)** A mutation at the splice acceptor site in Intron7 resulted in two mutant transcripts: one mutant had an out-of-frame deletion of the entire exon8, resulting in a premature translation termination signal, whereas the other mutant had an in-frame insertion of part of intron8 in addition to the deletion of the entire exon8, resulting in the deletion of 22 amino acids, which were replaced by 19 mutant amino acids. Exon–exon and intron–exon junctions are indicated by red dotted lines. Exons are not drawn to scale. E, exon; I, intron; F, forward primer; R, reverse primer.

To determine the effects of *ODAD1* mutations on the resulting transcripts, RT-PCR and Sanger sequencing were performed to detect *ODAD1* cDNA in bronchial mucosal biopsy samples collected from patient Ⅲ:1 and three control individuals. The primer set (Exon7F + 9R) showed that the patient had two different amplification products (Figure 2B). Sanger sequencing of the smaller amplification product (indicated by the red arrow) showed that all 68 bp of exon 8 of this variant was deleted, causing a premature termination signal [Figure 2D (middle); Table 1]. Sanger sequencing of the larger amplification product (indicated by the black arrow) showed overlapping peaks, and the main peaks could not be determined. Therefore, the amplification fragment was cloned into the pB-CAG-Amp vector to construct a recombinant plasmid containing an *ODAD1* cDNA fragment, which was identified by Sanger sequencing (Figure 2C). The results of the sequencing revealed two transcripts. One had a deletion of all 68 bp of exon8 and an insertion of 59 bp of intron8, leading to an in-frame deletion of 22 amino acids that were replaced by 19 mutant amino acids [Figure 2D (bottom); Table 1], whereas the other had the same sequence as the control individual. This may be due to the patient being a compound heterozygote or the mutation being leaky. The two aberrant transcripts (Mutant 1 and Mutant 2) were consistent with the high delta score (DS) for the variant sequence c.598-2A>C (acceptor loss: 0.96).

The c.71-2A>C was predicted by SpliceAI to result in an acceptor loss (AL: 0.68) in Intron 3. It has also been reported that the c.71-2A>C mutation in *ODAD1* could lead to PCD (Guo et al., 2020). However, we and previous investigators were unable to verify how c.71-2A>C alters splicing by RT-PCR or RNA-sequencing (RNA-seq). In the future, if we can obtain samples from humans or animals carrying the c.71-2A>C mutation in *ODAD1*, we will perform in-depth studies to verify this result.

### 3.3 Tertiary structure of ODAD1 variants

**FIGURE 3:**
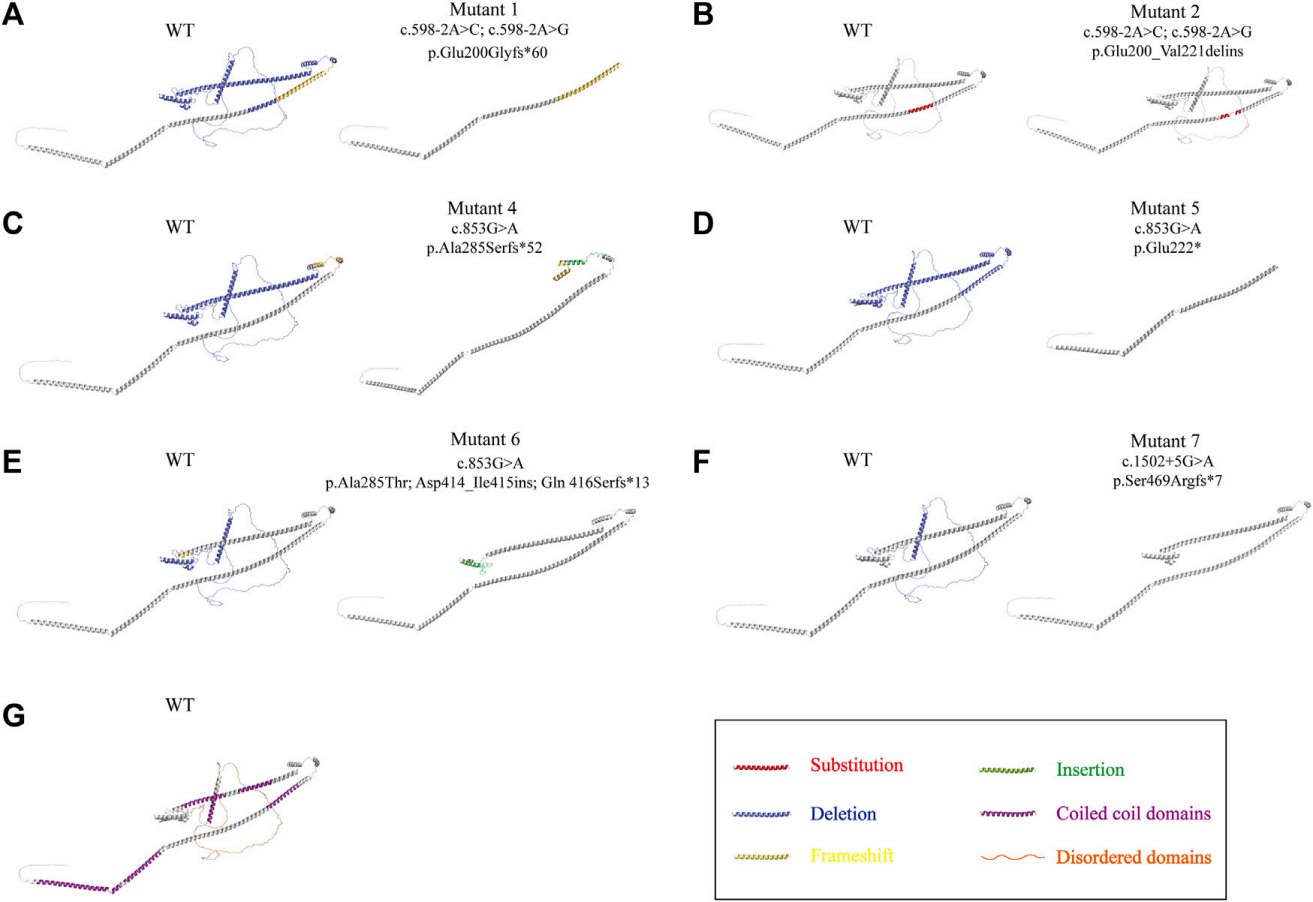
Alterations in the three-dimensional structure of ODAD1 variants. **(A)** Mutant1 had a deletion of the entire exon8, resulting in the frameshift mutation of exon9 and a premature translation termination signal. **(B)** In Mutant2, 22 aminoacids encoded by exon8 were replaced by 19 mutant aminoacids encoded by intron8. **(C)** The 285th alanine residue of mutant4 was replaced by serine. Mutant4 had an insertion of 26 mutant aminoacids encoded by intron9, resulting in a premature translation termination signal. **(D)** Mutant5 had an insertion of 98 bp of intron8, resulting in a frameshift mutation and premature translation termination signal at the end of exon8. **(E)** The 285th alanine residue of mutant 6 was replaced by threonine. In addition, 43 mutant aminoacids encoded by intron12 were inserted, resulting in the frameshift mutation of exon12 and a premature translation termination signal. **(F)** Mutant 7 had a deletion of 32 aminoacids encoded by exon14, resulting in the frameshift mutation of exon15 and a premature translation termination signal. Red, substitution; yellow, frameshift mutation; blue, deletion; green, insertion. **(G)** The wild-type ODAD1 contains six coiled-coil domains and three disordered domains. Purple, coiled-coil domain; orange, disordered domain.

The template-based homology modeling approach was used to investigate the effects of *ODAD1* splice-site mutations on the tertiary structure of the ODAD1 protein. We predicted the tertiary structures of six ODAD1 variants: two (mutants 1, 2) discovered in this study and four (mutants 4-7) reported in previous studies (Knowles et al., 2013; Onoufriadis et al., 2013; Ostrowski et al., 2022) (Figure 3; Supplementary Table S2). In Mutant2, 22 amino acids encoded by exon8 were replaced by 19 mutant amino acids encoded by intron8. However, all normal coiled-coil and disordered domains were retained (Figures 3B, G). Because each of the other six mutant transcripts had a premature translation termination signal, they all produced corresponding truncated proteins. The structure of the first five coiled-coil domains was completely conserved in Mutant6 and Mutant7 (Figures 3E, F). Mutant4 and Mutant5 retained the first three and first two coiled-coil domains, respectively (Figures 3C, D). Mutant1 retained the complete structure of the first two coiled-coil domains and had a frame-shift mutation in the third coiled-coil domain (Figure 3A). In the event that the complete tertiary structure of human ODAD1 wild-type and mutant proteins cannot be fully analyzed at present, modeling based on RNA transcripts can help us understand protein structure and function.

### 3.4 The *ODAD1* mutation (c.71-2A>C; c.598-2A>C) led to the absence of wild-type ODAD1 and the defects of the outer dynein arm in ciliary axonemes and caused a decrease in ciliary beat frequency

**FIGURE 4:**
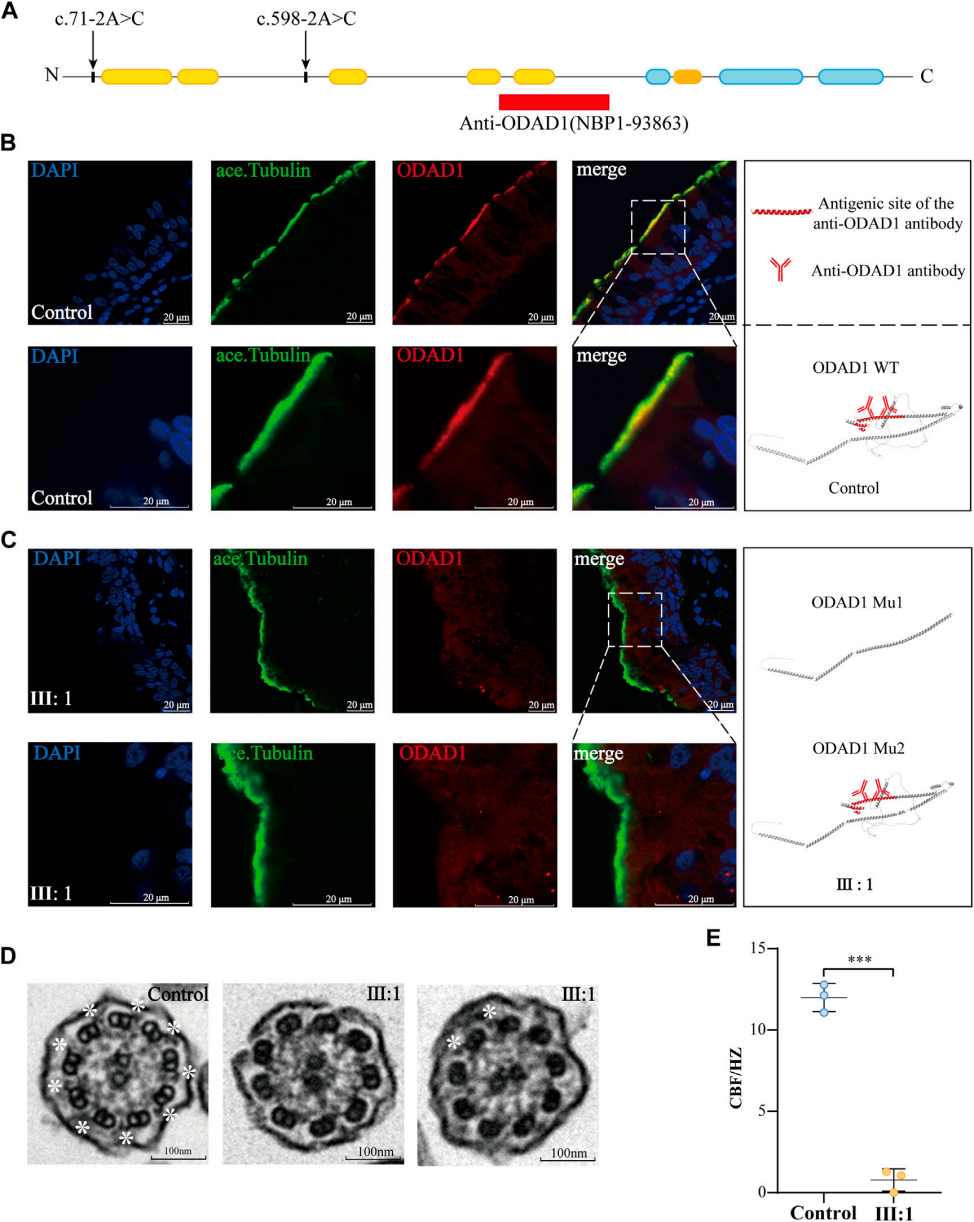
The *ODAD1* mutation (c.71-2A > C; c.598-2A > C) led to the absence of wild-type ODAD1 and the defects of the outer dynein arm in ciliary axonemes and caused a decrease in ciliary beat frequency. **(A)** Diagram of *ODAD1* demonstrating the antigenic site of the antibody used (red box) and the location of the mutation (arrow) in the patient. Yellow boxes represent predicted coiled-coil domains, and blue boxes represent disordered domains. **(B)** In control individuals, ODAD1 was localized along the length of the axoneme in ciliated cells. **(C)** In patient Ⅲ:1, the expression of ODAD1 was significantly low. Green, acetylated-α-tubulin; red, ODAD1; blue, DAPI. Scale bar = 20 μm. **(D)** Ultrastructure of the ciliary axonemes of the patient and control individuals was analyzed via TEM; defects in the ODA of ciliary axonemes were observed in the patient. While many ciliary cross-sections did not show ODA, some sections exhibited ODAs. The white asterisks indicate the structure of ODAs (scale bar = 100 nm). **(E)** CBF is significantly lower in the patient than in control individuals (***, *p* < 0.001).

To further investigate the effects of *ODAD1* mutations (c.71-2A>C or c.598-2A>C) on the expression of ODAD1, immunofluorescence microscopy was performed. *ODAD1* mutations in patient Ⅲ:1 resulted in the expression of two mutant proteins (Mutant1 and Mutant2), and one protein retained all normal coiled-coil domains (Mutant2) (Figures 3A, B; Figures 4B, C). Therefore, an anti-ODAD1 antibody (NBP1-93863) was used to detect proteins that retained the first five coiled-coil domains (Figure 4A). ODAD1 localized throughout the entire length of ciliary axonemes in control individuals (Figure 4B); however, its expression was considerably downregulated in patient Ⅲ:1 (Figure 4C).

To investigate the effects of *ODAD1* mutations (c.71-2A>C; c.598-2A>C) on the structure and function of the cilia, TEM and HSVA were performed. TEM of bronchial biopsy specimens from patient Ⅲ:1 demonstrated that most axonemes had no clear ODA, while several axonemes had two or more ODAs (Figure 4D; Supplementary Table S1). In addition, HSVA showed that CBF (0.78 ± 0.56 Hz) was significantly lower in patient Ⅲ: 1 than in control individuals (11.99 ± 0.70 Hz) (Figure 4E; Supplementary Videos S1–S3). However, contrary to what we expected and what had been found in previous studies in *ODAD1* (Onoufriadis et al., 2013; Guo et al., 2020), the cilia of patient III: 1 were not completely immobile. Although the CBF of the patient III: 1 was significantly decreased, the cilia appeared to beat in a coordinated fashion and may provide some force for mucociliary clearance (Supplementary Video S3). These results indicated that protein variants resulting from the splice-site mutations in *ODAD1* may retain partial function.

These results demonstrated that the compound mutation in *ODAD1* (c.71-2A>C; c.598-2A>C) led to the absence of wild-type ODAD1 and the defects of the outer dynein arm in ciliary axonemes and caused a decrease in ciliary beat frequency.

### 3.5 Results of co-IP demonstrated that most ODAD1 variants interacted with ODAD3

**FIGURE 5:**
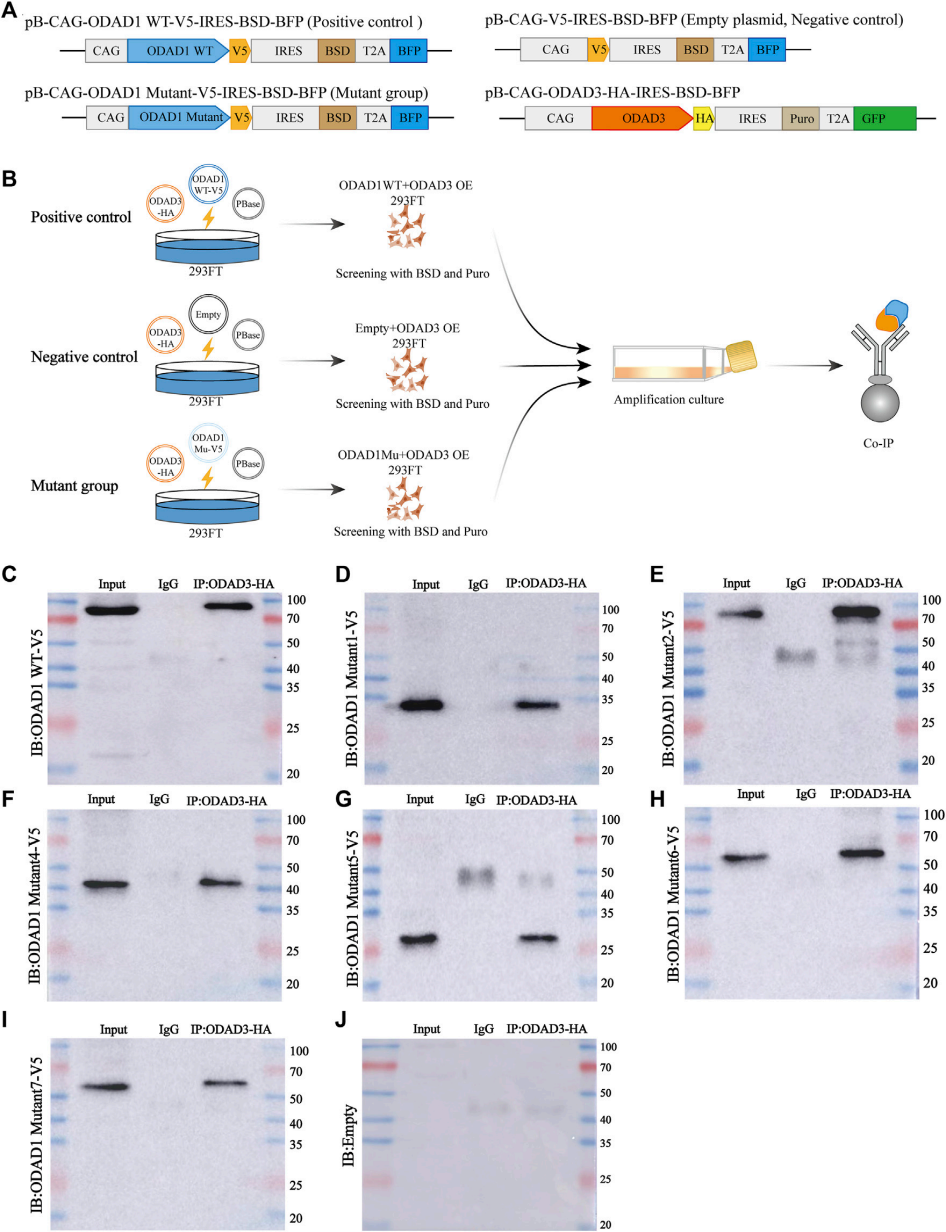
Co-IP results demonstrating the interaction between ODAD3 and most ODAD1 variants. **(A)** Genomic maps of plasmids used in this study. **(B)** Schematic illustration of the experimental design to evaluate the interaction between ODAD1 variants and ODAD3. Vector encoding C-terminal V5-tagged wild-type *ODAD1* or mutant *ODAD1* or empty plasmid was cotransfected with vector encoding C-terminal HA-tagged *ODAD3* into 293FT cells. HEK293FT cell lysates co-expressing V5-tagged wild-type *ODAD1*/mutant *ODAD1*/empty plasmid and HA-tagged *ODAD3* were immunoprecipitated with rabbit control IgG and rabbit anti-HA antibodies. The plasmid encoding wild-type ODAD1 was used as a positive control, whereas the empty plasmid was used as a negative control. **(C–J)** Immunoblotting with rabbit anti-V5 antibody showed that ODAD3 interacted with wild-type ODAD1 **(C)** and ODAD1 Mu1 **(D)**, Mu2 **(E)**, Mu4 **(F)**, Mu5 **(G)**, Mu6 **(H)**, and Mu7 **(I)**. WT, wild type; Mu, mutant; BSD, blasticidin; Puro, puromycin; IRES, internal ribosome entry site; BFP, blue fluorescent protein; GFP, green fluorescent protein; Co-IP, co-immunoprecipitation.

To investigate the functional role of ODAD1 variants in the assembly of ODAs, we examined the interaction between ODAD1 variants and ODAD3, a subunit of ODA-DC that is responsible for axonemal microtubule attachment of the ODAs. The results revealed that wide-type ODAD1 could interact with ODAD3 (Figure 5C). Therefore, it was used as a positive control, and an empty plasmid as a negative control to investigate whether the proteins resulting from splice-site mutations in *ODAD1* interact with ODAD3. All mutants that retained the first two coiled-coil domains were found to interact with ODAD3 (Figures 5C–J). These results indicate that the truncated proteins resulting from splice-site mutations in *ODAD1*, which retained the first two coiled-coil domains, could retain partial function.

### 3.6 ODAD1 mutants retaining the first three or more coiled-coil domains inhibited the interaction between wild-type ODAD1 and ODAD3

Ostrowski et al. (2022) had found that truncated proteins resulting from the *ODAD1* mutation (c.1502 + 5G>A) retain partial function but are insufficient to maintain normal ciliary function. Furthermore, (Chhin et al., 2009) restored cilia beating in human airway epithelial cells with *DNAI1*-mutaiton (c.48 + 2_48+3insT; c.1543G>A) using a lentiviral vector. However, the cilia beat in an uncoordinated manner. And the number of ODA per axoneme was significantly lower than normal, which may be due to some cells not being infected with the virus or to the endogenous mutant protein having a competitive inhibitory effect on the exogenous wild-type protein during dynein arm assembly. Based on the above results, we ask the following question: in gene therapy of PCD patients with *ODAD1* mutations, do dysfunctional endogenous mutant proteins inhibit the function of exogenous wild-type proteins?

**FIGURE 6:**
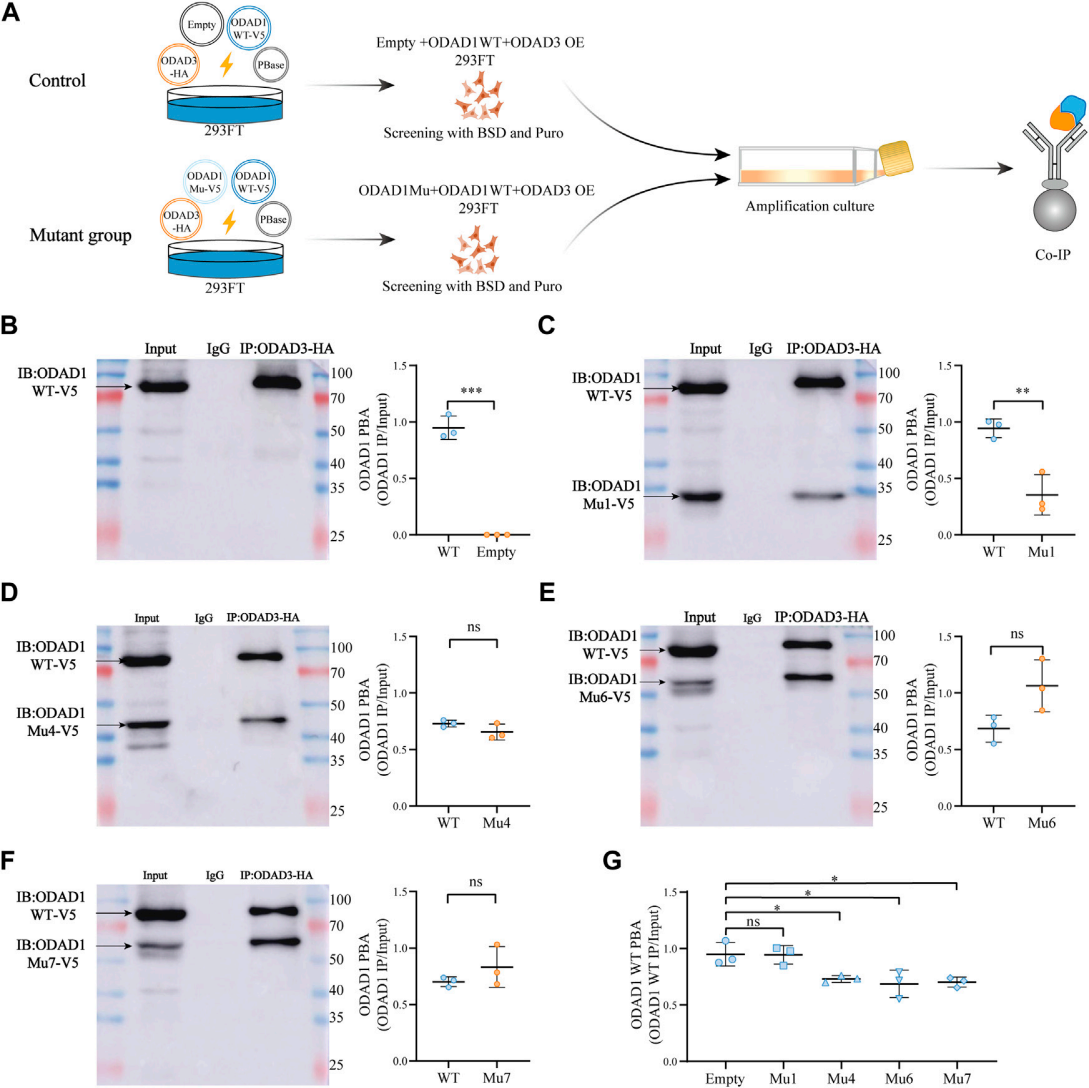
ODAD1 mutants retaining the first three or more coiled-coil domains inhibited the interaction between wild-type ODAD1 and ODAD3. **(A)** Schematic illustration of the experimental design to evaluate the interaction between ODAD1and ODAD3. Vector encoding C-terminal V5-tagged mutant *ODAD1* or empty plasmid was cotransfected with vector encoding C-terminal V5-tagged wild-type *ODAD1* and HA-tagged *ODAD3* into 293FT cells. HEK293FT cell lysates co-expressing V5-tagged wild-type *ODAD1*, mutant *ODAD1*/empty plasmid and HA-tagged *ODAD3* were immunoprecipitated with rabbit control IgG and rabbit anti-HA antibodies. The empty plasmid was used as a control. The total amount of each plasmid was 2 μg. For immunoblotting, the loading amount of Input was 20μg, and the volume of IgG and IP was 12 μL. **(B–F)** Immunoblotting with rabbit anti-V5 antibody showed that the protein-binding ability (PBA) of mutant 1, which retained the first two coiled-coil domains, for ODAD3 was weaker than that of wild-type ODAD1. However, the PBA of other truncated ODAD1 proteins (Mutant4, Mutant6, and Mutant7), which retained the first three or more coiled-coil domains, for ODAD3 was not different with that of wild-type ODAD1. **(G)** The truncated ODAD1 proteins, which retained the first three or more coiled-coil domains (Mu4, Mu6, and Mu7), resulted in a reduced interaction between wild-type ODAD1 and ODAD3. The expression of wild-type ODAD1 was not different between groups (Supplementary Figure S2). WT, wild type; Mu, mutant; BSD, blasticidin; Puro, puromycin; Co-IP, co-immunoprecipitation; PBA, protein-binding ability (PBA = IP/Input); NS, no significance. ***, *p* < 0.001; **, *p* < 0.01; *, *p* < 0.05.

Therefore, a co-immunoprecipitation assay was performed to determine whether proteins resulting from splice-site mutations in *ODAD1* affect the interaction between wild-type ODAD1 and ODAD3 in 293FT cells (Figure 6A). The results showed that the protein-binding ability (PBA) of Mutant1, which retained the first two coiled-coil domains, for ODAD3 was weaker than that of wild-type ODAD1 (Figures 6B, C). However, the PBA of other truncated ODAD1 proteins (Mutant4, Mutant6, and Mutant7), which retained the first three or more coiled-coil domains (Figures 6D–F), for ODAD3 was not different with that of wild-type ODAD1. Co-transfection of *ODAD1* mutants resulting in proteins retaining the first three or more coiled-coil domains inhibited the interaction between wild-type ODAD1 and ODAD3 under the condition that no difference was observed in the expression of wild-type ODAD1 between groups (Figure 6G; Supplementary Figure S4). These results suggested that ODAD1 mutants retaining the first three or more coiled-coil domains inhibited the interaction between wild-type ODAD1 and ODAD3.

## 4 Discussion

This study demonstrated that the compound mutation in *ODAD1* (c.71-2A>C; c.598-2A>C) led to aberrant splicing that resulted in the absence of the wild-type ODAD1 and defects of the outer dynein arm in ciliary axonemes, causing a decrease in ciliary beat frequency. Furthermore, we demonstrated that the truncated proteins resulting from splice-site mutations in *ODAD1* could retain partial function and inhibit the interaction between wild-type ODAD1 and ODAD3.

To date, 13 likely pathogenic mutation sites of *ODAD1* have been reported (Knowles et al., 2013; Onoufriadis et al., 2013; Li et al., 2019; Chen et al., 2020; Guo et al., 2020; Alsamri et al., 2021; Guan et al., 2021; Hannah et al., 2022; Ostrowski et al., 2022); however, only 3 sites have been demonstrated to result in corresponding mRNA changes and cause PCD (Knowles et al., 2013; Onoufriadis et al., 2013; Ostrowski et al., 2022), and the effects of the remaining 10 sites on the transcription of *ODAD1* remain unclear. In this study, we identified a compound heterozygous mutation in *ODAD1* (c.71-2A>C; c.598-2A>C) in a patient with PCD. The c.598-2A>C is a novel mutation that could result in two mutant transcripts (Mutant1 and Mutant2). These results are consistent with those of a study by Knowles et al. (Ostrowski et al., 2022). However, the mutation reported in the study by Knowles et al. (2013). was c.598-2A>G, which is different from that identified in this study. These results suggested that different base mutations at the same site in *ODAD1* could produce the same mutant transcripts.

To determine the effects of the c.71-2A>C mutation on the transcripts, primer sets (Exon 1F + Exon 5/9R and Exon 3F + Exon 9R) were designed. In both the patient and controls, the primer set (Exon 1F + Exon 5/9R) didn't show the expected amplification fragments. In the patient, the primer set (Exon 3F + Exon 9R) identified the third mutant transcript with a complex structure in which exon 5 was replaced by a partial exon structure of “9, 6, 7” (results not shown). We were unable to obtain nasal brushing, bronchial brushing, or bronchial mucosa biopsy specimens from the patient’s heterozygous parents or other individuals with the same mutation site. And to the best of our knowledge, there is no data regarding the possible transcript from the c.71-2A>C mutation. Therefore, although SpliceAI showed that c.71-2A>C weakened the canonical acceptor site in Intron 3, we were not certain whether the third aberrant transcript resulted from c.71-2A>C. In addition, we could not completely rule out that the complex structure may be the result of spurious priming or recombination during the amplification steps. (Wai et al., 2022). reported that short-amplicon RT-PCR was a useful alternative for analyzing splicing events in genes with low median transcripts per million (TPM) in blood RNA for clinical diagnosis. It was consistent with our results that the short-amplicon RT-PCR was more sensitive than the long-amplicon RT-PCR in RNA analysis of bronchial mucosal biopsy tissues (results not shown). If we can obtain samples from humans or animals with *ODAD1* mutations in the future, we will perform further in-depth studies to verify this result.

Mutant2, which retained all coiled-coil and disordered domains, was not detected in the ciliary axonemes of patient Ⅲ:1 via immunofluorescence analysis as expected (Figure 4C). The reasons underlying this finding may be as follows: the expression of Mutant2 was too low to be detected; the mutant was metabolized too quickly; the mutant could not bind to transport-related proteins, therefore, was not transported to ciliary axonemes, although it retained most of its normal structure. The specific reasons underlying this finding should be investigated in detail.

To understand the interaction among the components of the complex apparatus that produce synchronized cilia beats, it is important to visualize the three-dimensional (3D) structures of the cilia and the position of ciliary components relative to each other. In 2021, (Gui et al., 2021), reported the first systematic study on ODA-DC in mammalian cilia. They used cryo-ET to construct an atomic model of pentameric ODA-DC from bovine respiratory cilia. Unlike the trimeric ODA-DC (consisting of DC1, DC2, and DC3) of Chlamydomonas reinhardtii, the bovine pentameric ODA-DC consists of ODAD1 (CCDC114), ODAD2 (ARMC4), ODAD3 (CCDC151), ODAD4 (TTC25), and ODAD5 (Calaxin). In addition, previous studies have suggested that axonemal localization of ODAD1 depends on ODAD3 (Hjeij et al., 2014), and that of ODAD2 depends on ODAD1 (Hjeij et al., 2013). The assembly of ODA-DC and ODA on the axoneme is interdependent and synergistic; however, the underlying mechanism remains unclear (Ma et al., 2019; Gui et al., 2021; Kubo et al., 2021).

To investigate the effects of *ODAD1* splice-site mutations on the tertiary structure and function of the ODAD1 protein, we used SWISS-MODEL to predict the tertiary structures of six ODAD1 variants: two (mutants 1,2) identified in this study and four (mutants 4-7) reported in previous studies (Knowles et al., 2013; Onoufriadis et al., 2013; Ostrowski et al., 2022). To the best of our knowledge, this study is the first to report the tertiary structure of ODAD1 variants resulting from splice-site mutations. In addition, we examined the interaction between ODAD1 variants and ODAD3 through Co-IP assay. The truncated proteins resulting from *ODAD1* splice-site mutations, which retained the first two or more coiled-coil domains, retained partial function and interacted with ODAD3. These results explain why the cilia of patient Ⅲ:1 were not completely immobile as expected. (Ostrowski et al., 2022). had found that the *ODAD1* mutation (c.1502+5G > A) led to the expression of truncated proteins attached to the axoneme, which allowed for the assembly of some ODAs and a significant level of ciliary activity. However, the immunostaining of ODAD1 showed that most of the truncated proteins were localized in the proximal region of the axoneme. And while some sections had two or more ODAs, many ciliary cross-sections showed no ODA. Although the subject’s cilia were not completely immobile, the beating frequency was significantly reduced compared with the control. Besides, the subject had situs inversus totalis, a frequent productive cough, and a few episodes of bronchitis. These results suggest that the truncated proteins resulting from the *ODAD1* mutation retain partial function but are insufficient to maintain normal cilia function.

PCD is a rare genetic disease with complex clinical expression and without definitive treatment. Gene therapy is an encouraging alternative treatment to conventional drugs that have been inefficient up to now. Gene replacement therapy is the most widely used method in studies of PCD gene-based therapy. The first and, to date, only study to restore the ciliary function of airway epithelial cells in patients with PCD by overexpressing exogenous genes was conducted by Chhin and coworkers in 2009 (Chhin et al., 2009). Cilia cells were collected from a patient with PCD harboring a heterozygous compound *DNAI1* mutation (c.48 + 2_48+3insT; c.1543G > A). Overexpression of wild-type *DNAI1* in these cells cultured *in vitro* recovered CBF; however, cilia beating was uncoordinated, and the number of ODA per axoneme was significantly lower than normal. This could be due to the fact that some cells were not infected with the virus or that the intracellular mutant proteins have a competitive inhibitory effect on the exogenous wild-type protein during the assembly of the dynein arms. This is also consistent with our findings that ODAD1 mutant proteins retaining the first three or more coiled-coil domains significantly inhibit the interaction between wild-type ODAD1 and ODAD3. We could not verify these results in human ciliary cells because of the difficulty in obtaining sufficient human samples to construct an adequate model of pseudostratified ciliated columnar epithelium.

In conclusion, this study expands the mutational and clinical spectrum of PCD, provides more evidence for genetic counseling and individualized treatment and offers novel insights into the development of gene therapy for PCD. In addition to selecting appropriate vectors to overexpress wild-type proteins *in vivo*, reducing or eliminating inhibitory effects of endogenous mutant proteins may improve therapeutic efficacy.

## Data Availability

The data presented in the study are deposited in the China National Genebank (CNGB, https://db.cngb.org/cnsa/) repository, accession number CNP0004603.
