# Supplementary material for: ODAD1 variants resulting from splice-site mutations retain partial function and cause primary ciliary dyskinesia with outer dynein arm defects
Source: Front Genet. 2023 Oct 31;14:1270278. doi: 10.3389/fgene.2023.1270278 (PMC10651219; doi:10.3389/fgene.2023.1270278)
Supplement: Supplementary file 2 [file DataSheet1.pdf]

Supplementary Material

1 Supplementary Data

Figure S1: The proband had typical characteristics of PCD;

Figure S2: Transcription of *ODAD1* (NM_001364171.2) was detected in bronchial mucosa biopsy samples and peripheral blood cells;

Figure S3: Splicing prediction for the *ODAD1* mutations (c.71-2A>C; c.598-2A>C);

Figure S4: The expression of wild-type ODAD1 was not different between groups.

Table S1: Demographic and clinical characteristics of a patient with PCD and her family members;

Table S2: Effect of *ODAD1* mutations on cDNA transcript in individuals with PCD;

Table S3: Primer sequences used in RT-PCR;

Table S4: Primer sequences used for Sanger sequencing.

Video S1-Control: Video showing ciliary activity in control.

Video S2-Proband: Video showing ciliary activity in the patient III:1.

Video S3-Proband: Video showing ciliary activity in the patient III:1.

2 Supplementary Figures and Tables

2.1 Supplementary Figures

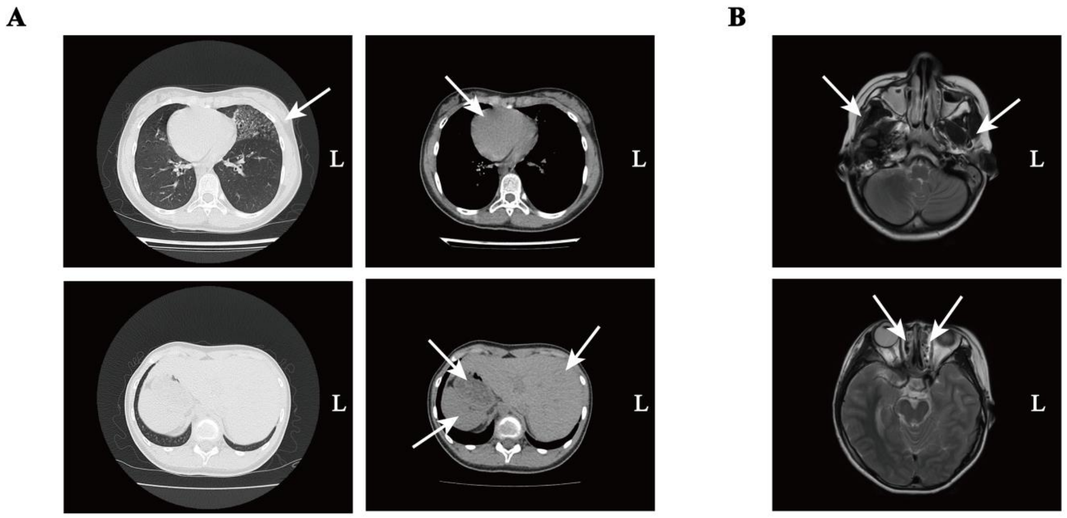

Supplementary Figure 1. The proband had typical characteristics of PCD. (A) Chest computed tomography (CT) showing bronchiectasis (left) and situs inversus (right). The white arrow reflects bronchiectasis (left top), dextrocardia (right top and bottom), and abdominal organ heterotaxy: the liver on the left side; the stomach and spleen on the right side (right bottom). (B) Magnetic resonance imaging (MRI) of the nasal sinuses shows pansinusitis. The white arrow reflects nasosinusitis.

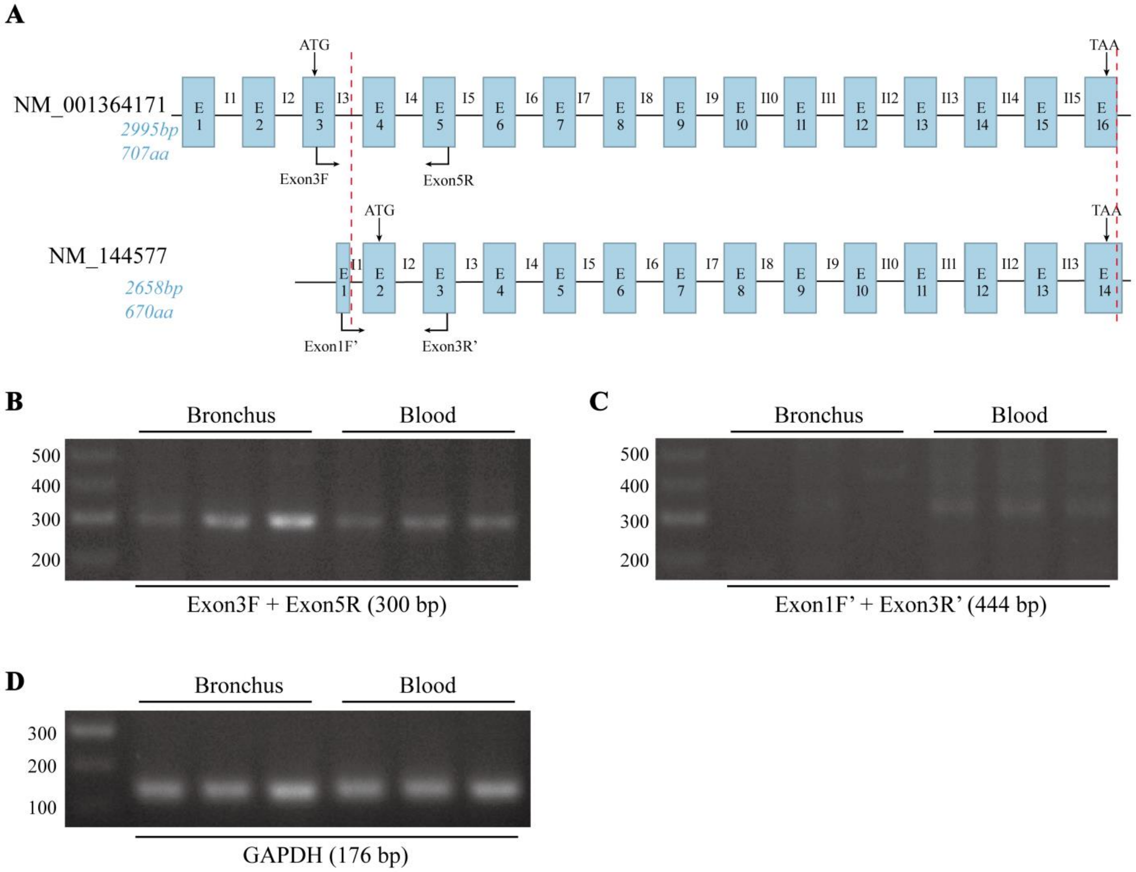

Supplementary Figure 2. Transcription of *ODAD1* (NM_001364171) was detected in bronchial mucosa biopsy samples and peripheral blood cells. (A) Structural similarities and differences between the two transcripts of *ODAD1*. The transcript NM_001364171 consists of 2995 bp and contains 16 exons encoding a protein of 707 amino acids. The transcript NM_14457 consists of 2658 bp and contains 14 exons encoding a protein with 670 amino acids. The base sequences between the red dashed lines are identical. Start and stop codons are indicated. Introns/exons are not drawn to scale. E, exon; I, intron; F, forward primer; R, reverse primer. (B) Primer Exon3F was designed to match the transcript NM _001364171 and not to match the transcript NM _144577. The primer set (Exon3F + Exon5R) showed the expected 300 bp amplification fragment in bronchial mucosal biopsy specimens and peripheral blood mononuclear cells. (C) Primer Exon1F' was designed to matches the NM_144577 and doesn't match the NM_001364171. In both bronchial mucosal biopsy specimens and peripheral blood mononuclear cells, the primer set (Exon1F' + Exon3R') didn't show the expected 444 bp amplification fragment. (D) *GAPDH* as a positive control showed the expected 176 bp amplification fragment. Samples were from 6 different individuals. F, forward primer; R, reverse primer.

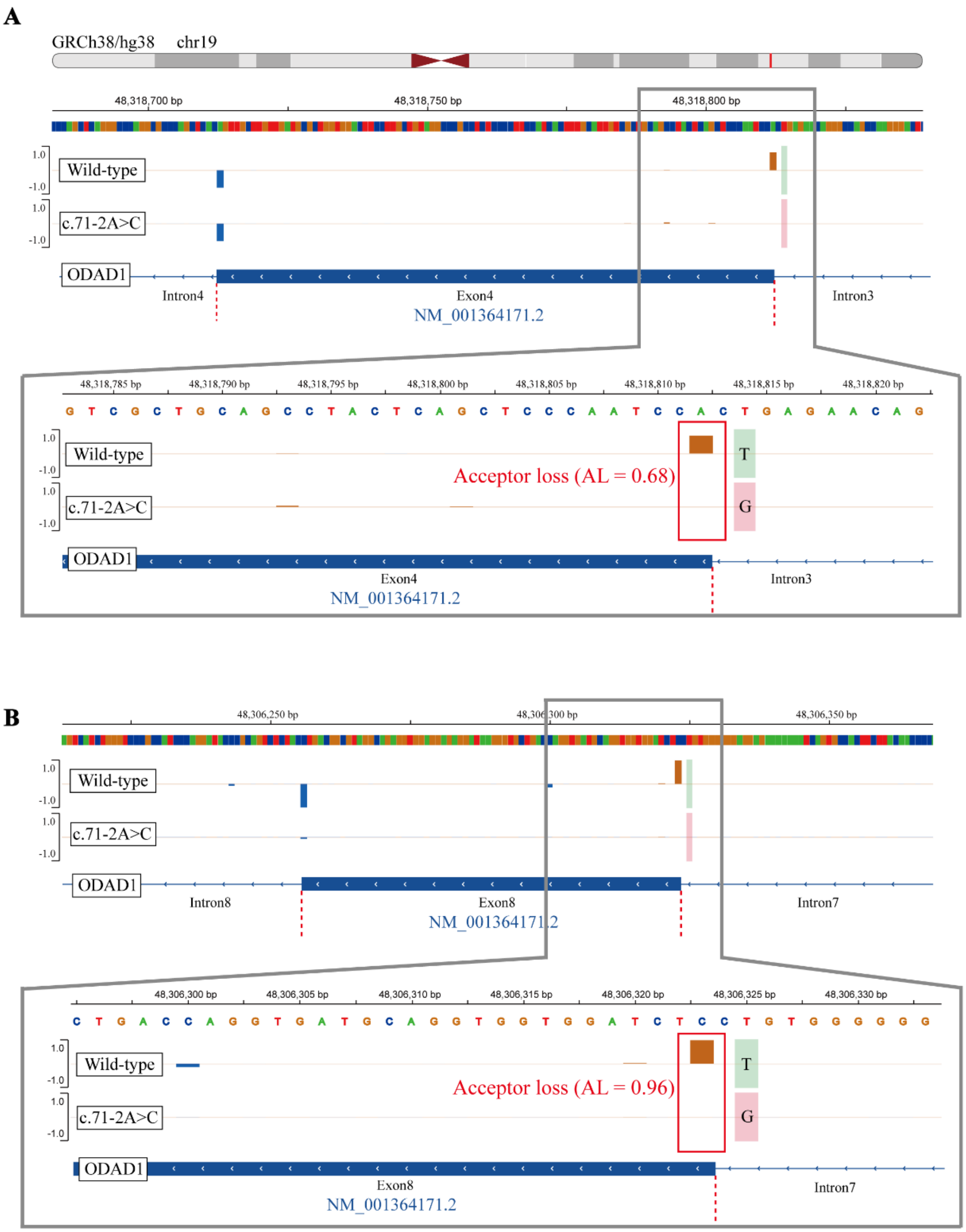

Supplementary Figure 3. Splicing prediction for the *ODAD1* mutations (c.71-2A>C; c.598-2A>C). (A) c.71-2A>C weakened the canonical acceptor site in Intron 3 (acceptor loss of 0.68). (B) c.598-2A>C weakened the canonical acceptor site in Intron 7 (acceptor loss of 0.96). Orange bar: acceptor site prediction ([0,1]); Blue bar: donor site prediction ([-1,0]). Green and red highlights show the variation sites on the wild-type and mutant track, respectively.

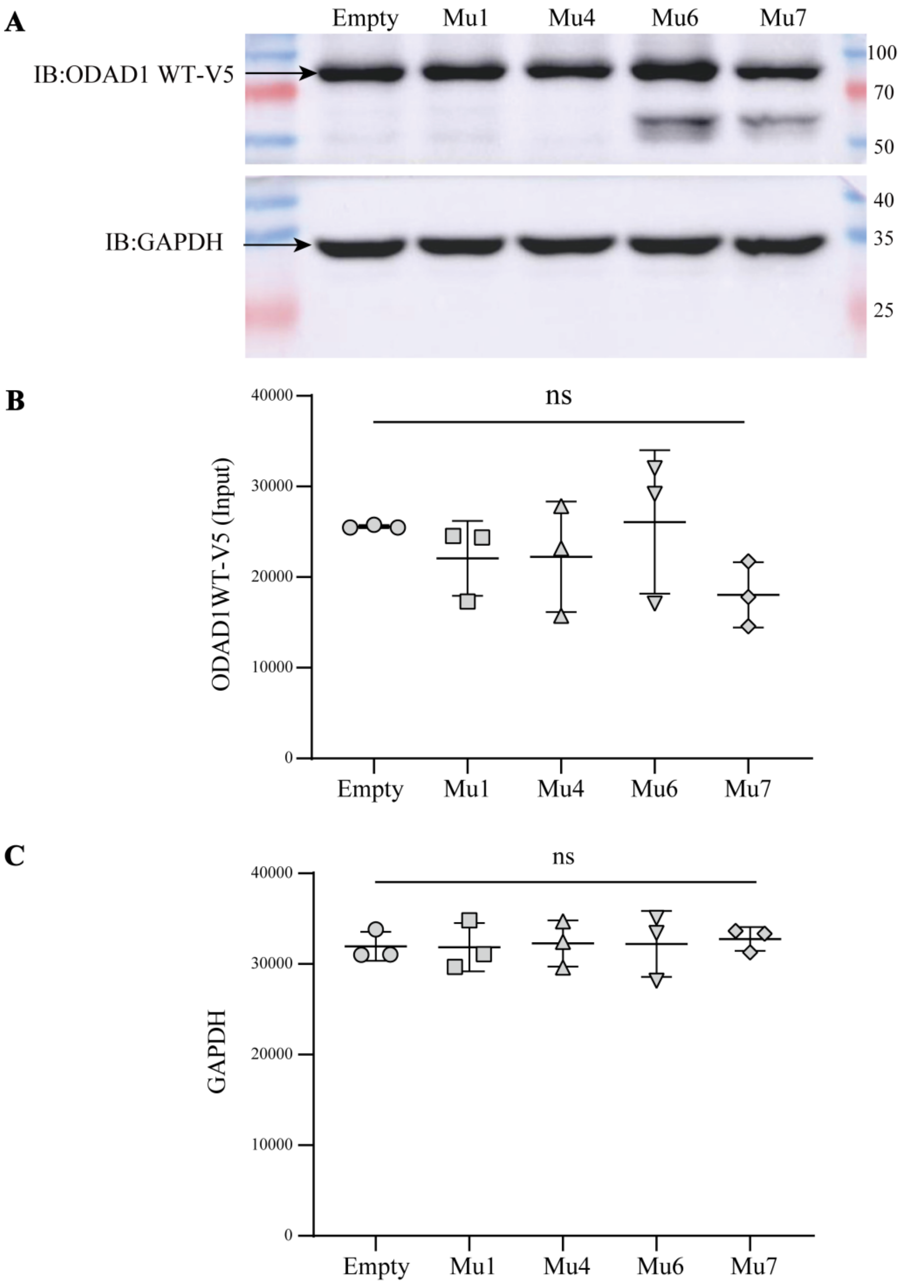

Supplementary Figure 4. The expression of wild-type ODAD1 was not different between groups. Vector encoding C-terminal V5-tagged mutant *ODAD1* or empty plasmid was cotransfected with vector encoding C-terminal V5-tagged wild-type *ODAD1* and HA -tagged *ODAD3* in 293FT cells. The empty plasmid served as a control. The total amount of every plasmid was 2 µg. Immunoblotting with rabbit anti-V5 antibody showed that the expression of wild-type ODAD1 was not different between groups. *GAPDH* served as a reference. In every group, the loading amount of Input was 20µg, and the volume of IgG and IP was 12 µL. WT, wild type; Mu, mutant; NS, no significance.

2.2 Supplementary Tables

Supplementary Table 1. Demographic and clinical characteristics of a patient with PCD and her family members

ID	Ethnicity	Age (years)	CHD	Neo RDS	Chronic wet cough	Bronchi-ectasis	Situs inversus	Sinusitis	Otitis Media	Pulmonary Function			nNO (nL/min)[a]	TEM	CBF (HZ)	Fertility defect
										FVC %Pred	FEV_1 %Pred	FEV_1/FVC				
I:1	Asian	71	No	No	No	No	No	No	No	NA	NA	NA	NA	NA	NA	No
I:2	Asian	70	No	No	No	No	No	No	No	NA	NA	NA	NA	NA	NA	No
I:3	Asian	67	No	No	No	No	No	No	No	NA	NA	NA	NA	NA	NA	No
I:4	Asian	67	No	No	No	No	No	No	No	NA	NA	NA	NA	NA	NA	No
II:1	Asian	40	No	No	No	No	No	No	No	NA	NA	NA	NA	NA	NA	No
II:2	Asian	38	No	No	No	No	No	No	No	NA	NA	NA	NA	NA	NA	No
II:3	Asian	41	No	No	No	No	No	No	No	NA	NA	NA	NA	NA	NA	No
II:4	Asian	35	No	No	No	No	No	No	No	NA	NA	NA	NA	NA	NA	No
III:1	Asian	12	No	No	Yes	Yes	Yes	Yes	Yes	55%	42%	75%	5.4	ODA	0.77	Untested; too young
III:2	Asian	8	No	No	No	No	No	No	No	NA	NA	NA	NA	NA	NA	Untested; too young

[a] PCD-specific cut-off level <77 nL/min.

Abbreviations: CHD, congenital heart disease; Neo RDS, neonatal respiratory distress; FVC, forced vital capacity; FEV_1, forced expiratory volume in the first second; NA, not available;nNO, nasal nitric oxide; TEM, transmission electron microscopy; ODA, outer dynein arms; CBF, ciliary beat frequency.

Supplementary Table 2. Effect of *ODAD1* mutations on cDNA transcript in individuals with PCD

Mutant	Genomic Mutations			Primer Set[a]	cDNA Transcript after RT-PCR	Predicted Amino Acid Change	Study	Comments
	Transcript	Exon/ Intron	Base Change					
**Mutant 1**	NM_001364171	Intron 7	c.598-2A>C	Exon 7 F + 9 R	r.598_665del	p.Glu200Glyfs*60	Current	Mutant 1 had deletion of all 68 bp of exon 8 followed by joining of exons 7 and 9. Wild-type amplifification product:365 bp. Mutant amplifification product: 297 bp.
	NM_001364171	Intron 7	c.598-2A>G	Exon 6 F + 12 R	r.598_665del	p.Glu200Glyfs*60	Knowle et al (Knowles et al., 2013)	
**Mutant 2**	NM_001364171	Intron 7	c.598-2A>C	Exon 7 F + 9 R	r.598-1_598-59ins; 598_665del	p.Glu200_Val221delins	Current	Mutant 2 had insertion of 59 bp from the end of intron 8 as well as deletion of all 68 bp of exon 8 leading to in-frame deletion of 22 amino acids that are replaced by insertion of 19 mutant amino acids. Wild-type amplifification product:365 bp. Mutant amplifification product: 356 bp.
	NM_001364171	Intron 7	c.598-2A>G	Exon6 F + 12 R	r.598-1_598-59ins; 598_665del	p.Glu200_Val221delins	Knowles et al (Knowles et al., 2013)	
**Mutant 4**	NM_001364171	Exon 9	c.853G>A	Exon 6 F + 12 R Exon 8 F + 10 R	r. 853g>a; 853+1_853+79ins	p.Ala285Serfs*52	Knowles et al (Knowles et al., 2013) Onoufriadis et al (Onoufriadis et al., 2013)	Mutant 4 had insertion of 79 bp from start of intron 9 followed by cryptic splice donor site and addition of exon 8.

**Mutant 5**	NM_001364171	Exon 9	c.853G>A	Exon 6 F + 12 R	r. 665+1_665+98ins; 853+1_853+79ins	p.Glu222*	Knowles et al (Knowles et al., 2013)	Mutant 5 had insertion of 98 bp from start of intron 8 followed by cryptic splice donor site and addition of exon 9, as well as insertion of 79 bp from start of intron 9 followed by cryptic splice donor site and addition of exon 10.
**Mutant 6**	NM_001364171	Exon 9	c.853G>A	Exon 12 F + 15 R	r. 853g>a; 1240+1394_1240+1521ins	p.Ala285Thr; Asp414_Ile415ins; Gln416Serfs*13	Knowles et al (Knowles et al., 2013)	Mutant 6 had insertion of 128 bp from the middle of intron 12 and addition of exon13.
**Mutant 7**	NM_001364171	Intron 14	c.1502+5G>A	Exon 12 F + 16 R	r. 1405_1502del	p.Ser469Argfs*7	Knowles et al (Knowles et al., 2013) Ostrowski et al (Ostrowski et al., 2022)	Mutant 7 had deletion of all 98 bp of exon 14 followed by joining of exons 13 and 15.

[a]Primer sequences in this study were listed in Table S3. RT-PCR was carried out on biopsy tissues of human bronchial mucosa.
Abbreviations: F, forward primer; R, reverse primer; NA, not available.

Supplementary Table 3. Primer sequences used in RT-PCR

**Gene**	**Mutation in Genomic DNA**	**Transcript**	**Primer Location**	**Base Sequence in 5'-3' Direction[a]**	Restriction Enzyme Sites	**Wild Type Amplification**
ODAD1	c.598-2A>C	NM_001364171	Exon7-F	CGACGCGTCACCTGTCACTTTGACAACCAGCTG	MluI	380bp
			Exon9-R	CGGAATTCGCTTTTCACGCTTCTCCAGGACATC	EcoRI	
ODAD1	c.71-2A>C/ c.598-2A>C	NM_001364171	Exon3-F	CCGCTCGAGGAGGGAAGCGAGGCATTTCTGGAG GGAAT	XhoI	796bp
			Exon9-R	CGGAATTCGGCTGCCGGTCGTTGTTCTTGAG	EcoRI	
ODAD1	NA	NM_001364171	Exon3-F	GAGGGAAGCGAGGCATTTCTGGAGGGAAT	NA	300bp
			Exon5-R	CTGCTCCTGCAGCTCCTCGATCTC	NA	
ODAD1	NA	NM_144577	Exon1-F'	CTGCAGTGAGCTGAGATTGTGCCACTGCGCTAGA	NA	444bp
			Exon3-R'	CTGCTCCTGCAGCTCCTCGATCTC	NA	
GAPDH	NA	NM_001289745	Exon8-F	CATCAAGAAGGTGGTGAAGCAG	NA	176bp
			Exon8/9-R	TGTCATACCAGGAAATGAGCTT	NA	
ODAD1	NA	NM_001364171	Exon3-F	CGACGCGTCCACCATGCCTTTGGGACGCTTGGCA GGGAG	MluI	2199bp
			Exon16-V5-R	ATAAGAATGCTAGCACCGGTTTATGTGGAGTCCA GGCCCAGCAGAGGGTTAG	NheI	
ODAD1 Mutant 2	c.598-2A>C	NM_001364171	Exon9-F	CAGCCTCCAGTCATCCTGACCCCGGCTGACCCCC GCAGGGAGGAGGCGAAGGCCAAGATGGGCTTG	NA	10171bp

			Exon9-R	GATGACTGGAGGCTGGACTGGGGTCGGCCTGGG ATCCTTCTTCAGCTTGCGGTCCACGTTC	NA	
ODAD1 Mutant 5	c.853G>A	NM_001364171	Exon3-F	CGACGCGTCCACCATGCCTTTGGGACGCTTGGCA GGGAG	MluI	688bp
			Exon8-R	CCGCTCGAGCCTGACGGCGTAGGCAGAGGTGGA GGAG	XhoI	
ODAD1 Mutant 7	c.1502+5G>A	NM_001364171	Exon3-F	CGACGCGTCCACCATGCCTTTGGGACGCTTGGCA GGGAG	MluI	1444bp
			Exon13/14-R	CCGCTCGAGAAACCCGGGGGGTCTTCTCTGGGC ATGTAGGAAGGCCTGCACTG	XhoI	
ODAD3	NA	NM_145045	Exon1-F	CGACGCGTCCACCATGACATCTCCTCTGTGCAGG GCGGCCTC	MluI	1806bp
			Exon13-R	GAAGATCTGGACCTCCGAGAGCGACGGTGCTTC TTGTG	BglII	

[a]Red is the restriction enzyme site.
Abbreviations: F, forward primer; R, reverse primer; NA, not available.

Supplementary Table 4. Primer sequences used for Sanger sequencing

Genomic/Plasmid	Primer Location	Base sequence in 5’-3’ direction
ODAD1 (NM_001364171)	Exon4-R	GTCCACCAGCGCATCAACAA
	Inron8-R	GTCCACCAGCGCATCAACAA
pB-CAG-*ODAD1*cDNA-Amp	LTR-R	GATGAGCATATCCTCTCTGC
pb-CAG-*ODAD1* WT-V5-IRES-BSD-T2A-BFP<br>pb-CAG-*ODAD1* Mutant-V5-IRES-BSD-T2A-BFP	CAG-F	CCTGGGCAACGTGCT
	ODAD1-BglII-F	CAGGTCCTGCAGCGGCAGATCTTGCAC
	IRES-R	CGACCTTATTCCAAGCGGCT
pb-CAG-*ODAD3*-HA-IRES-Puro-T2A-GFP	CAG-F	CCTGGGCAACGTGCT
	ODAD3TF-2	GACGAGAGCCTCAACTTGGAG
	IRES-R	CGACCTTATTCCAAGCGGCT

Abbreviations: F, forward primer; R, reverse primer.

3 References

Knowles M. R., Leigh M. W., Ostrowski L. E., Huang L., Carson J. L., Hazucha M. J. et al. (2013). Exome sequencing identifies mutations in CCDC114 as a cause of primary ciliary dyskinesia. Am J Hum Genet. 92**,** 99-106.

Onoufriadis A., Paff T., Antony D., Shoemark A., Micha D., Kuyt B. et al. (2013). Splice-site mutations in the axonemal outer dynein arm docking complex gene CCDC114 cause primary ciliary dyskinesia. Am J Hum Genet. 92**,** 88-98.

Ostrowski L. E., Yin W., Smith A. J., Sears P. R., Bustamante-Marin X. M., Dang H. et al. (2022). Expression of a Truncated Form of ODAD1 Associated with an Unusually Mild Primary Ciliary Dyskinesia Phenotype. Int J Mol Sci. 23**,** 1753-1770.
